# Single-cell RNA-Seq reveals intracellular microbial diversity within immune cells during SARS-CoV-2 infection and recovery

**DOI:** 10.1016/j.isci.2023.108357

**Published:** 2023-10-30

**Authors:** Sunita Yadav, Priyanka Mehta, Jyoti Soni, Partha Chattopadhyay, Priti Devi, Thierry Habyarimana, Kishore Tardalkar, Meghnad Joshi, Rajesh Pandey

**Affiliations:** 1 Division of Immunology and Infectious Disease Biology, INtegrative GENomics of HOst-PathogEn (INGEN-HOPE) Laboratory, CSIR-Institute of Genomics and Integrative Biology (CSIR-IGIB), Mall Road, Delhi 110007, India; 2 Academy of Scientific and Innovative Research (AcSIR), Ghaziabad 201002, India; 3 Dr. D. Y. Patil Medical College, Hospital and Research Institute, Kolhapur, Maharashtra 416003, India; 4 Department of Biomedical Laboratory Sciences, INES-Ruhengeri, Ruhengeri, Rwanda

**Keywords:** Immunology, Virology

## Abstract

Intracellular microorganisms, like viruses, bacteria, and fungi, pose challenges in detection due to their non-culturable forms. Transcriptomic analysis at cellular level enables exploration of distributions and the impact of these microorganisms on host cells, a domain that remains underexplored because of methodological limitations. Single-cell technology shows promise in addressing this by capturing polyadenine-tailed transcripts, because recent studies confirmed polyadenylation in microbial transcriptomes. We utilized single-cell RNA-seq from PBMCs to probe intracellular microbes in healthy, SARS-CoV-2-positive, and recovered individuals. Among 76 bacterial species detected, 16 showed significant abundance differences. *Buchnera aphidicola*, *Streptomyces clavuligerus*, and *Ehrlichia canis* emerged significantly in memory-B, Naïve-T, and Treg cells. *Staphylococcus aureus*, *Mycoplasma mycoides*, *Leptospira interrogans*, and others displayed elevated levels in SARS-CoV-2-positive patients, suggesting possible disease association. This highlights the strength of single-cell technology in revealing potential microorganism’s cell-specific functions. Further research is essential for functional understanding of their cell-specific abundance across physiological states.

## Highlights

• 76 bacterial species of 15 genera across healthy, COVID-19-positive, and recovered individuals
• Higher abundance of opportunistic intracellular bacteria in recovered group
• Higher microbial abundance within antigen-presenting cells of COVID-19 patients
• Differential microbial abundance in Treg, memory T, and platelets in COVID-19

## Graphical abstract

**Figure:**
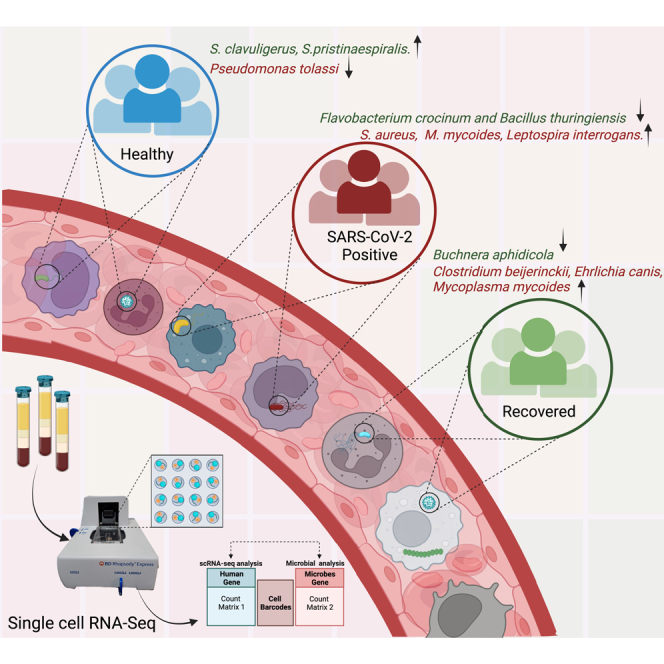

## Introduction

The ambiguous nature of microorganisms, spanning a spectrum from bacteria and viruses to fungi, persists owing to their pivotal role in sculpting the host’s immune response. Depending on their innate traits or, occasionally, the local environmental conditions, these bacteria have the ability to serve as commensal, pathogenic, or opportunistic agents. Precisely deciphering the interaction between microbes and the host’s immune response is pivotal for understanding their impact on the severity/outcome of the infectious disease.^1^ Various studies have shown the role of upper and lower respiratory tract microbial communities in modulating the severity of COVID-19 disease. For instance, bacteria like *Achromobacter xylosoxidan*s, *Staphylococcus aureus*, and *Mycoplasma pneumonia* were shown to be associated with COVID-19 mortality.^2^^,^^3^^,^^4^ Beyond COVID-19, comparable trends have been identified in other diseases, encompassing both acute infections like influenza and chronic infections like HIV (human immunodeficiency virus), MTb (*Mycobacterium tuberculosis*), and HCV (hepatitis C virus) where the initial infection disrupts the microbial balance aiding other microbes to thrive, creating an environment conducive to subsequent infections, leading to co-infections or superinfections.^5^^,^^6^

The presence of microbes, either intracellular or extracellular, significantly impacts how they interact with the host’s immune system and their capacity to evade immune defenses.^7^^,^^8^ Microbes preferentially interact with the host immune system at a close range due to the unique ecological niche within the host’s body. When microbes establish an infection, they often reside in close proximity to host tissues or cells, due to their need for nutrients, adaptation to evade immune defenses, transmission strategies, localized pathology, evasion of humoral immunity, immunomodulation, and complex life cycles.^9^^,^^10^ Intriguingly, many intracellular pathogens thrive within highly efficient antimicrobial defense cells, such as macrophages and dendritic cells. For example, *M. tuberculosis* and *S. aureus* can thrive within macrophages, wherein it resides within the phagosome by hijacking the phagosomal processes.^11^^,^^12^ Alternatively, intracellular microbes may inhabit specific host cell compartments such as the endosome or cytosol, where they secure vital nutrients and evade direct antibody attacks. In some cases, neutrophils, fibroblasts, or epithelial cells can also serve as habitat for intracellular pathogens.^12^^,^^13^ Gaining insights into the functionality of these microorganisms at the cellular level becomes crucial in tackling the complexities of treating these microbes.^14^

Next-generation sequencing (NGS) techniques, notably metatranscriptomics, metagenomics, and RNA sequencing (RNA-seq), are some of the important approaches traditionally used in unraveling the intricacies of host and pathogen genomes. This avenue of investigation not only unveils crucial insights into disease mechanisms, evolution, and potential therapeutic avenues but also offers a comprehensive perspective on the interplay between hosts and pathogens at a molecular level.^15^^,^^16^^,^^17^ However, when it comes to studying the presence of intracellular microbes, the conventional techniques tend to provide an average genome output that can obscure crucial insights.^18^ In contrast, single-cell sequencing offers a unique and powerful window into the complex world of individual cells, particularly in the context of studying microbial presence in the blood PBMCs. This technology enables the identification of rare cell types and allows us to understand how individual cells respond differently to various conditions. When applied to microbial analysis, single-cell sequencing uncovers new facets of cellular functions and interactions within the context of the host immune system.

Traditionally, single-cell technologies have primarily focused on capturing RNA based on the polyA tail, which was thought to be exclusive to eukaryotic cells. However, recent studies have overturned this assumption, revealing that numerous viruses such as the influenza virus, SARS-CoV-2, and duck hepatitis A, along with many bacteria including *Escherichia coli* and *Bacillus subtilis*, possess polyA tails in their mRNA. This discovery opens an exciting avenue for studying the presence of these microbes in the blood PBMCs at a single-cell resolution. By harnessing this approach, researchers can delve into the cell-specific abundance and function of microbes within the intricate landscape of the host immune system, shedding light on how they interact, evade defenses, and potentially contribute to disease.^19^^,^^20^

In this study, we delve into the complex interplay of microbes within specific cell types in PBMCs. This investigation centered on microbial communities within PBMCs from three groups: healthy individuals, SARS-CoV-2-positive individuals, and those who have recovered. The aim was to identify and elucidate the diversity of intracellular microbes found within the definitive cell types using scRNA-Seq technology and to uncover potential associations between microbial presence and host cellular responses at different physiological conditions. Toward that, we identified 76 microbes enriched within 12 distinct immune cell types. Of these, 16 microbes were differentially abundant between different across the healthy, positive, and recovered groups. A closer look at the cell-type wise abundance of these microbes revealed 8 differentially expressed bacterial species across the groups in 6 different cell types.

## Results

### Differential presence of non-human reads in healthy, SARS-CoV-2-positive, and recovered individuals

**Figure 1:**
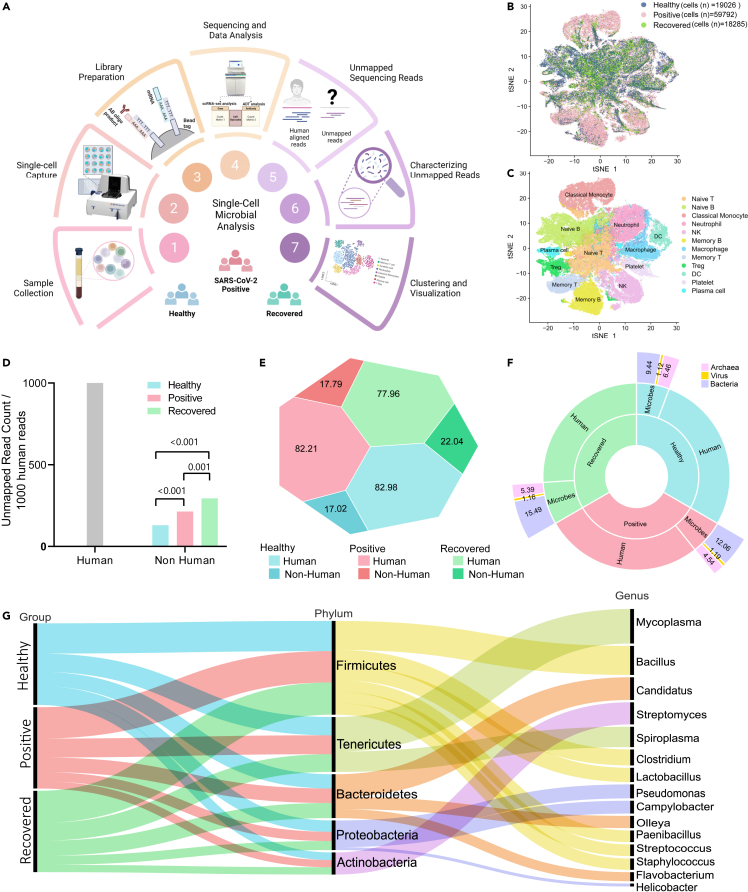
Differential presence of non-human reads in scRNAseq of PBMC of healthy, SARS-CoV-2-positive and recovered individuals (A) Graphical representation of a single-cell-based study, designed to investigate the diversity of microbes present within the immune cells of healthy, SARS-CoV-2-positive and recovered individuals. (B) tSNE plot represents the distribution of cells in the three groups—healthy (blue), positive (pink), and recovered (green). (C) Annotation of the clusters into 12 specific cell types. (D) The bar plot represents the number of unmapped read counts per thousand human reads across different groups—healthy (blue), positive (pink), and recovered (green); data represented as mean. (E) The proportion plot illustrates the distribution of human versus non-human reads within each group; data represented as percentage (%). (F) The sunburst plot displays the distribution of microbial reads identified through kraken analysis, categorizing them into archaeal, viral, and bacterial reads within the non-human reads; data represented as percentage (%). (G) The alluvial plot represents the top genus and their corresponding phyla.

In this study, we employed a single-cell multiomics dataset from the PBMCs of healthy, COVID-19-positive, and recovered individuals generated by our group using microwell-based BD Rhapsody platform.^21^ We selected 27 individuals (14 COVID-19-positive, 10 recovered, and 3 healthy) based on the clinical parameters for understanding cell-type-specific microbial abundance (Figure 1A, Table S1). Bioinformatic analysis was performed using Seurat R 4.2 package to correct for any batch effects, filter out low-quality reads, and perform unsupervised clustering of cells. A total of 97103 cells were captured post filtration—19,026 from healthy individuals; 59,792 from positive; and 18,285 from recovered individuals (Figure 1B). We identified 12 different cell clusters, viz. naive T cell, naive B cell, classical monocytes, neutrophil, natural killer (NK) cell, memory B cell, macrophages, memory T cell, Treg cell, dendritic cells (DC), platelets, and plasma cells across the three groups (Figure 1C). A total of ∼11 billion reads were generated from 97,103 cells, of which ∼9 billion reads mapped to the human genome, whereas nearly 2 billion reads remained unmapped. The differential proportions of human and non-human reads found in each group were as follows: healthy (82.98% human versus 17.02% non-human), positive (82.21% human versus 17.79% non-human), and recovered (77.96% human versus 22.09% non-human), wherein we identified a significant differential distribution of non-human reads per 1000 human reads across the three groups (p value < 0.001) (Figures 1D and 1E).

To discern the nature of these non-human reads, whether they were a result of sequencing artifacts or not, we used the Kraken2 tool for taxonomic classification of these sequences. Our analysis revealed that these sequences belonged to microbial entities contributed predominantly by bacteria across the three groups, constituting 9.44%, 12.06%, and 15.49% of the reads in the healthy, COVID-19-positive, and recovered, respectively. Subsequent to bacteria, archaea were identified at proportions of 6.46%, 4.54%, and 5.39% in the corresponding groups. Additionally, viruses were detected, comprising 1.12%, 1.19%, and 1.16% of the reads in the respective groups (Figure 1F). We filtered out microbial species having less than 0.1% abundance and without annotated reference genomes to identify the most abundant phyla. We identified the presence of 5 phyla, i.e., Proteobacteria (24.7%, 25%, and 24.6%), Firmicutes (18%, 18.5%, and 19%), Bacteroidetes (10.5%, 10.5%, and 10.8%), Actinobacteria (8.3%, 7.8%, and 8.1%), and Tenericutes (7.5%, 7.8%, and 7.4%) within the respective groups (Figure 1G, Table S2). Overall, we identified a total of 76 bacterial species across the 15 genera and 3 groups, which were further analyzed at single-cell resolution for their cell-type-specific abundance and species-specific functions across the three groups.

### Differential abundance of bacterial species in COVID-19-infected and recovered individuals versus healthy individuals

**Figure 2:**
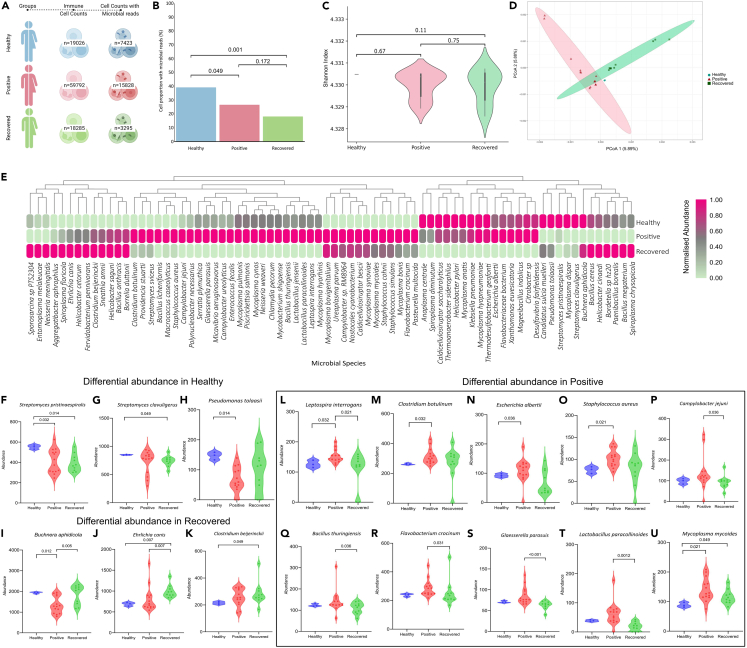
Differential presence of microbial species at single-cell level across healthy, positive, and recovered (A) Distribution of total cells captured and subset of cells with substantial microbial presence across the healthy, positive, and recovered. (B) The bar plot represents the proportion of cells with more than 5 microbial reads between the healthy, positive, and recovered; data represented as mean. (C) Alpha diversity analysis shows no significant difference of microbial diversity between the healthy, positive, and recovered. (D) The principal coordinate analysis (PCoA) plot represents beta diversity and shows differential clustering between the three groups—healthy (blue), positive (red), and recovered (green). (E) The heatmap represents the top 76 microbial species. (F–H) The violin plots represent microbes that are differentially abundant in the healthy group with respect to positive and recovered. (I–K) Differentially abundant microbes in the recovered with respect to healthy and positive. (L–U) Microbes that are differentially abundant in the positive with respect to healthy and recovered.

In order to elucidate the pattern of microbial abundance at single-cell resolution, we filtered out cells with <5 microbial reads for at least one species. Post QC, we retained 26,546 cells with microbial reads out of the total 97,103 cells, wherein we observed a significant decrease in percentage of cells with microbial reads in the positive (26.47%) and recovered (18.02%) compared with the healthy (39.01%) (Figures 2A and 2B). Because all the 76 microbes were detected in the 3 groups, no significant difference in Shannon alpha diversity was observed (Figure 2C). However, our beta diversity analysis utilizing the Bray Curtis distance matrix unveiled distinctive cluster patterns, indicating that despite having the same number of microbial species, they are differentially abundant between the three groups (Figure 2D). Subsequent adonis tests were performed to validate pronounced dissimilarities of microbial abundance across the three groups (p value = 0.003).

When comparing the differential abundance of the 76 microbes, a distinct trend in abundance among the various groups became evident (Figure 2E). The abundance of a total of 16 bacteria was found to be significantly different in at least one comparison group (healthy versus positive, positive versus recovered, healthy versus recovered). In the healthy, higher abundance of *Streptomyces pristinaespiralis* (compared with positive and recovered), *S. clavuligerus* (compared with recovered), and *Pseudomonas tolaasii* (compared with positive) was observed (Figures 2F–2H). In the recovered group, we observed higher abundance of *Buchnera aphidicola*, *Ehrlichia canis* and *Clostridium beijerinckii* (compared with positive/healthy) (Figures 2I–2K). Conversely, *Leptospira interrogans*, *Mycoplasma mycoides*, *Clostridium botulinum*, *Escherichia albertii, S. aureus*, *Campylobacter jejuni*, *Bacillus thuringiensis*, *Flavobacterium crocinum*, *Glaesserella parasuis*, and *Lactobacillus paraclinoid* were found to be differentially abundant in the SARS-CoV-2-positive patients as compared with healthy and recovered (Figures 2L–2U).

An important finding from our analysis was that all the three bacterial species that are highly abundant in the recovered (*B. aphidicola*, *C. beijerinckii*, and *E. canis*) are opportunistic in nature.^22^^,^^23^^,^^24^ The increased abundance of opportunistic bacteria in the recovered might be attributed to the dysfunctional T cell response, as T cell dysfunction is associated with suppressed innate immune response.^25^^,^^26^ Intriguingly, *C. beijerinckii* and *E. canis* are reported to be associated with inflammatory response, which might be one of the contributory factors for the increased inflammatory response, post-recovery.^27^^,^^28^ The findings highlight the intricate and complex nature of host-microbe interplay during infection and recovery and importance of understanding the cell-type-specific microbial diversity. Further research is essential to explore the specific functions and potential implications of these bacteria in the context of infection, recovery, and overall health.

### Immune-cell-type-specific significant bacteria and their potential roles

**Figure 3:**
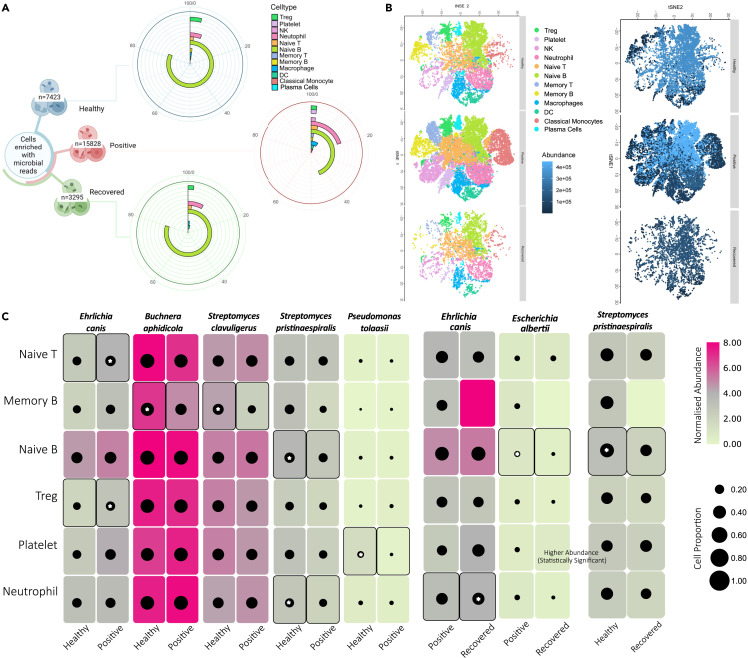
Differential abundance of microbes within specific immune cell types (A) The circular bar plot represents the abundance of microbes within the cell types across the groups; data represented as percentage (%). (B) tSNE plot showing the cell types with microbial reads across healthy, positive, and recovered. (C) The heatmap shows the abundance of microbial reads (with log2 fold change >0.5 and adjusted p value <0.05) between different groups (healthy-positive, positive-recovered, healthy-recovered) in more than 10% of cells. The size of the black dots represents the proportion of cells containing respective bacterial reads. The star (∗) between the black dots represent the significance in that group.

To elucidate the potential implications posed by these 16 bacterial species across the healthy, SARS-CoV-2-positive, and recovered individuals, we looked at their abundances across the 12 different cell types. Microbial presence was observed in 8 cell types in healthy, 12 cell types in positive, and 6 cell types in the recovered, whereas 6 cell types (Treg, neutrophil, naive T cells, naive B cells, macrophages, DC) exhibited microbial reads in all the three groups, suggesting their consistent presence across different health conditions. Interestingly, all these 6 cell types are involved in the antigen presentation process (Figure 3A). Our analysis revealed that although naive T and Treg cells have similar relative abundance across the three groups, microbial abundance within these cells across the three groups are strikingly different (Figure 3B). Although higher microbial abundance in the antigen-presenting cells is in line with the function of these cells, differential microbial abundance in cell types such as platelets, memory T, and Treg needs closer attention for functional relevance. Additionally, we observed microbial abundance in the classical monocyte, NK cell, and plasma cells exclusively in the SARS-CoV-2-positive patients. Although classical monocytes and NK cells are known to be involved in the antigen presentation process, the role of plasma cells during COVID-19 needs context-specific exploration/elucidation (Table S3).

Upon investigating significant bacteria across different cell types within their respective groups, we identified six bacteria—namely *E. canis*, *B. aphidicola*, *S. clavuligerus*, *S. pristinaespiralis*, *P. tolaasii*, and *E. albertii*—as being of importance at the cellular level. Remarkably, these significant bacteria exhibited a consistent pattern with what was observed at the group level after applying log2 fold change and other filter criteria (Figure 3C). This consistency suggests that the trends identified at the group level hold true and are translated to the cellular level. Of particular interest is *E. canis*, an intracellular bacterium, which was found to be significantly enriched in the naive T cells and Treg of the positive patients compared with the healthy individuals. Additionally, it was observed to be more abundant in neutrophils of the recovered compared with the positive. This suggests a plausible unique role of *E. canis* in the T-cell-mediated immune response of COVID-19-positive patients. Since it is known to cause apoptosis in monocytes, the role of this bacteria in T cell biology and function could be explored in the future.^28^ On the other hand, bacterial species like *B. aphidicola*, *S. clavuligerus*, *S. pristinaespiralis*, and *Pseudomonas tolaasii* were significantly enriched in the memory B cells, naive B cells, neutrophils, and platelets of the healthy individuals compared with the positive patients. Moreover, *S. pristinaespiralis* was found in the naive B cells of the healthy group compared with the recovered as well, whereas *E. albertii* was observed to be enriched in the naive B cells of the positive patients compared with the recovered. The presence of specific microorganisms within specific cell types plausibly suggests an interaction between the host and the differential abundance of the microbes. Going forward, further research is required to grasp the underlying biological mechanisms at play.

This study unveils intriguing differences in microbial reads at the cellular level between healthy, positive, and recovered individuals. The identification of specific bacterial species associated with different cell types sheds light on the intricate interactions between the immune system and microbes during various health states and recovery processes. These findings hold potential for further research, and validating these results further will be essential to fully understand the role of these significant bacteria in immune cell interactions and their overall impact on health and disease outcomes.

### Converging functional insights: comparative analysis of microbial species across the bulk RNA-Seq and single-cell RNA-Seq

**Figure 4:**
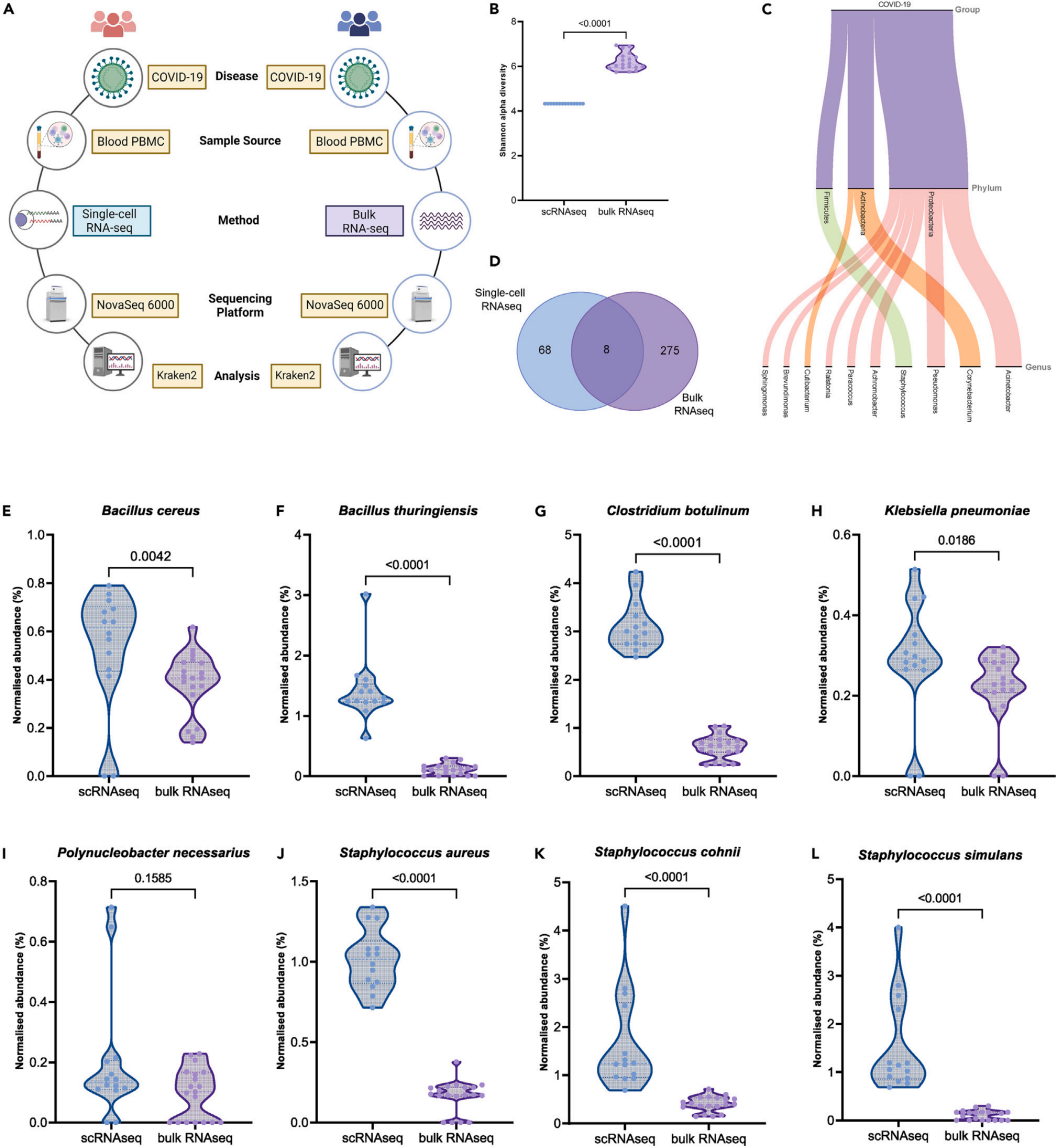
Validation of scRNAseq-based findings using a complementary technique in a different cohort (A) Although the disease, sample source, sequencing platform, and analysis methods are constant between the two cohorts used for detection of microbial reads, they differ only with respect to the underlying capturing technique. (B) The comparison of alpha diversity between scRNAseq and bulk RNAseq cohorts. (C) The alluvial plot represents the top genus and their corresponding phyla in the bulk RNA-Seq data. (D) The Venn diagram shows unique bacterial species found in the bulk and scRNA-Seq as well as overlap between the two. The violin plot represents the comparison of abundances proportion between scRNAseq and bulk RNAseq COVID-19-positive cohort of the 8 common species: (E) *Bacillus cereus*, (F) *Bacillus thuringiensis*, (G) *Clostridium botulinum*, (H) *Klebsiella pneumoniae*, (I) *Polynucleobacter necessarius*, (J) *Staphylococcus aureus*, (K) *Staphylococcus cohnii*, and (L) *Staphylococcus simulans.*

To enhance the broader applicability and validate specific significance of our single-cell level study, we additionally assessed microbial profiles from 19 COVID-19 patients’ blood PBMC from a different cohort through bulk RNA-Seq. A comparison of the clinical and demographic parameters of the two cohorts is presented in Table 1. Although both the samples were sequenced using Illumina NovaSeq 6000 platform, 101 paired-end reads from COVID-19-positive patients’ blood PBMC, they only differed based on their capturing method (Figure 4A). These patients were admitted to the ICU at ID and BG Hospital in Kolkata, India.^29^ Upon comparing the Shannon alpha diversity between single-cell RNAseq and bulk RNAseq data, we observed a significantly higher estimate of microbial species in the latter cohort, which may be due to the underlying whole transcriptome bulk RNAseq method (Figure 4B). We further looked into the phylum and genus levels, aiming to discern potential discrepancies in the COVID-19 patients. Notably, 10 genera (with >0.1 relative abundance) were captured in bulk RNAseq data, of which 2 genera (Staphylococcus and Pseudomonas) overlapped with scRNAseq COVID-19 patient group (Figure 4C, Table S4). Overall, we identified a total of 283 bacterial species in bulk RNAseq samples (Figure 4D). Among them, 7 species demonstrated an overlap with the cohort of 76 microbial species found at single-cell level: *Bacillus cereus*, *B. thuringiensis*, *C. botulinum*, *Klebsiella pneumoniae*, *S. aureus*, *Staphylococcus cohnii*, and *Staphylococcus simulans*. Upon comparing the abundance of these 7 species between scRNAseq and bulk RNAseq data, we observed significant differences in proportions in all the species (Figures 4E–4L). Remarkably, 3 species—*C. botulinum*, *B. thuringiensis*, and *S. aureus*—demonstrated significant enrichment in the positive group in comparison to both the healthy and recovered groups (Figures 2M, 2O, and 2Q). This coherence reinforces the notable relevance of these specific species in the context of disease progression.

**Table 1:** Clinical characteristics comparison between scRNAseq and bulk RNA-seq COVID-19-positive patients

	scRNAseq COVID-19-positive (n = 14)	Bulk RNA-seq COVID-19-positive (n = 19)	p *value*
**Demographics**			

Age mean (IQR)	62 (53.5–66.5)	59 (51.5–74)	0.62^a^
Gender (M|F)	9|5	16|3	0.36^b^
HRCT score	0.54 (0.5–0.72)	NA	–
CRP	54 (1.63–75.3)	NA	–

**Comorbidities**			

Hypertension^c^	5 (35.71%)	10 (52.63%)	0.54^b^
Diabetes mellitus^c^	5 (35.71%)	9 (47.36%)	0.75^b^
Ct score	NA	23.37 (19.29–26.20)	–

Data represented as n(%) or n(IQR).

a Mann–Whitney U test.

b Chi square test.

c Missing data points.

## Discussion

The intricate interplay between the disease severity and presence of microbes within the immune cell types, shedding light on host immune responses during infectious diseases, remains a largely uncharted domain. The recent COVID-19 pandemic serves as a prime example, where a wide spectrum of disease severity was observed.^3^^,^^30^^,^^31^ This variability is attributed to factors such as host immune reactions, viral strain heterogeneity as well as co-presence of diverse microbial species.^32^^,^^33^ Leveraging on the scRNA-seq, we unveil the cellular landscape of peripheral blood mononuclear cells from healthy, positive, and recovered COVID-19 patients. This study pioneers the investigation of bacterial profiles within the immune cells at a single-cell resolution across different health states and augments important insights.

To date, ample evidence has been provided to understand the differential and dynamic immune response underlying individuals across healthy, recovered, and COVID-19-positive.^33^ Although the immune system guards against microbial intrusion to curtail infections, some microbes manage to infiltrate and persist within the body. Consequently, dysbiosis at different taxa levels and their niches in the host body can affect immunity and disease outcomes, underscoring the importance of investigating microbial presence and their impact on immune cell dysregulation. Of note, studies have revealed the prominence of common phyla (Proteobacteria, Actinobacteria, Firmicutes, and Bacteroidetes) in the immune cells of healthy individuals and those with respiratory conditions like asthma, with the noteworthy presence of related genera linked to disease severity in both asthma and COVID-19.^34^^,^^35^

In this line, we have observed no noteworthy distinctions in microbial composition concerning diversity, as well as at the levels of phylum and genus, among the healthy, positive, and recovered. Moreover, detailed insights at the species level remain limited and insufficiently explored. Noteworthy species such as *E. coli*, *Bacillus* sp., *Campylobacter hominis*, *Pseudomonas* sp., *Thermoanaerobacter pseudethanolicus*, *T. thermosaccharolyticum*, and *Staphylococcus epidermis* have shown positive correlations with COVID-19 severity and proinflammatory response.^35^^,^^36^^,^^37^ Delving deeper into our analysis, a comprehensive examination of 76 bacterial species unveiled a discernible trend involving 16 distinct species across the various groups. Notably, the positive and recovered groups exhibited an enrichment of potential pathogenic species, whereas the healthy individuals showcased a prevalence of specific commensal counterparts. Particularly intriguing were the findings related to *S. clavuligerus* and *S. pristinaespiralis*—both commensal inhabitants within the healthy individuals. These species, known for producing beneficial antibiotics, clavulanic acid and pristinamycin, could signify a role in strengthening immune defenses.^38^^,^^39^^,^^40^ In contrast, a decline in these commensal species was noted in the recovered and positive patients, potentially indicating a correlation with clinical conditions. Concurrently, among the identified species such as *S. aureus*, *M. mycoides*, *L. interrogans*, *G. parasuis*, *E. albertii*, and *C. jejuni* exhibited significant enrichment among the positive patients, distinguishing them from the healthy and recovered individuals. The presence of these microbes indicates the use of such immunomodulation strategies by these microbes during infection that may contribute to excessive inflammatory responses (cytokines), which can impair the immune system’s functioning.^41^^,^^42^ For example, *S. aureus*, functioning as a facultative intracellular pathogen, adroitly avoids opsonophagocytic through its distinct surface attributes. Once established, it thrives within phagosomes, escaping to the cytosol, thus initiating host cell apoptosis. This bacterium also stimulates host cell autophagy, extracting essential nutrients and energy for its metabolic sustenance.^41^^,^^42^ Significantly, *S. aureus* infections incite the host’s inflammatory response by surviving within (Polymorphonuclear neutrophils) PMNs, underscoring its virulence and potential for mortality, as corroborated by Gresham et al.^43^ Another notable instance is *L. interrogans*, which infiltrates monocytes and can lead to exaggerated inflammatory responses, impairing immune functionality.^44^^,^^45^^,^^46^^,^^47^ Similarly, *M. mycoides* trigger a respiratory burst in phagocytic cells, whereas *G. parasuis*, a pathogenic entity, induces reactive oxygen species, thereby upregulating proinflammatory cytokine production.^48^^,^^49^^,^^50^^,^^51^ Further adding to the complexity, the pathogenic bacterium *C. jejuni* stimulates both innate and adaptive immune responses, resulting in prolonged inflammation. Studies have confirmed its long-term survival and replication within monocytes, alongside lymphocyte apoptosis induction.^52^

**Figure 5:**
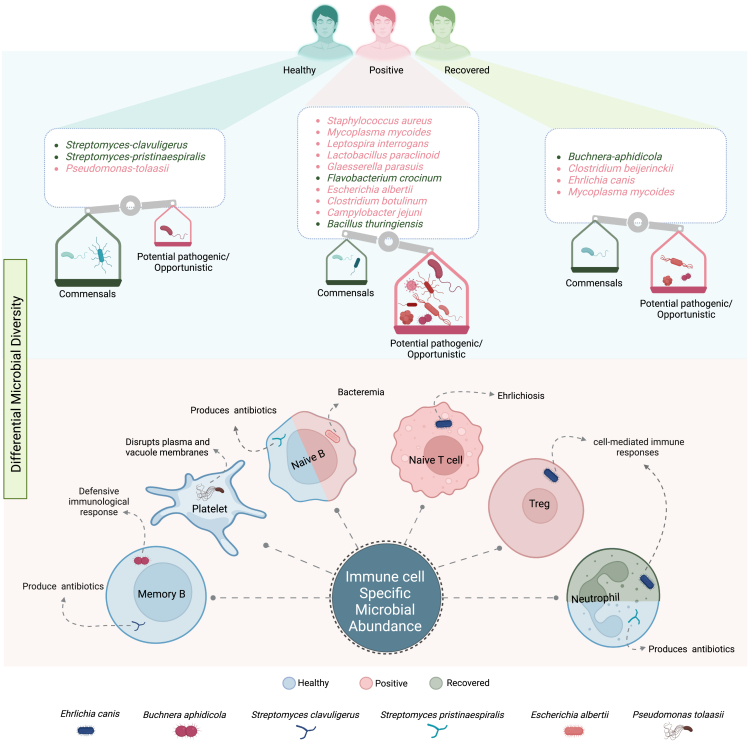
Summary of the study key findings (A) Group-level abundance and categorization of microbes presented a comprehensive analysis of microbial abundance across the different groups, categorizing them into commensal and pathogenic species. This assessment offered crucial insights into the microbial composition of the studied population. The bottom half represents specific immune cells that host a variety of microbial species and the potential roles of these microbes within the immune cell environment.

Similarly, certain bacteria exhibited increased prevalence in the recovered individuals, including *E. canis*, *B. aphidicola*, and *C. beijerinckii*. Although these species have been reported in disease contexts, their relevance to human infections requires further exploration. The consistent elevation in their abundance might align with the body’s response to infection and the subsequent recovery process. Overall, our observations revealed a discernible alteration in bacterial abundance, with a tendency toward opportunistic or potentially pathogenic bacteria in COVID-19-positive and recovered individuals, juxtaposed with a more pronounced prevalence of commensal microorganisms within the healthy group (Figure 5).

Notably, substantial bacterial diversity is observed across various blood components, including buffy coat, red blood cells, and plasma. Analyzing the cellular distribution of the aforementioned bacterial species in relation to the healthy, positive, and recovered individuals, we found significant microbial presence in only 6 out of the 12 cell types. Remarkably, *S. clavuligerus* and *S. pristinaespiralis* exhibited higher prevalence in memory B and naive B cells of healthy individuals, suggesting a potential reservoir role. Memory B cells are known for their long-lasting immune memory and long life. This contribution could foster harmonious microbiota persistence within a robust immune system. In a parallel observation, *E. canis* was more prevalent in both Tregs and naive T cells, whereas *E. albertii* displayed greater detection in the naive B cells among SARS-CoV-2-positive patients compared with the recovered individuals. This presence within the immune cells suggests pathogen evasion, given these cells’ role in infection defense and immune response activation. Interestingly, *P. tolaasii* was enriched in the platelet cells of healthy individuals compared with the positive patients, displaying a counterintuitive trend compared with the group-level pattern. However, whether *P. tolaasii* has a positive or neutral impact on human health, and if its potential involvement in apoptosis and membrane disruption might have different implications within the human microbiota compared with its effects in hosts, requires further research (Figure 5).

To validate our findings at the group level, we investigated an additional dataset of COVID-19 patients from a separate geographical region and discovered the presence of 8 bacterial species in the PBMCs, coinciding with our results. Although the validation dataset was generated by bulk RNA-seq, it shares four main similarities: the sequencing platform (Illumina NovaSeq 6000), read length (101 x 2), and underlying disease (COVID-19), and sample type (PBMC) with the scRNAseq dataset. Notably, within this overlap, 3 potential pathogenic species demonstrated significant enrichment in the positive patients within our cohort (Figures 4D–4K). This leads to a noteworthy observation that microbes can be found in PBMCs not only during infection but also in healthy and recovered individuals. Although the smaller overlap between the two datasets could be linked to regional distinctions, capturing resolution as well as capturing method (polyA-based capture in single-cell versus total RNA in bulk RNA-seq), the cross-platform data establish the fact that the microbes can reside within the PBMCs. Few other recent studies have also highlighted the presence of microbes in blood and in PBMCs. We also observed a strong cell-type-specific abundance of specific bacterial species as well as a shift in the overall species composition across the healthy, positive and recovered groups, albeit no significant difference in the alpha and beta diversity at the group level was observed in both the cohorts. This highlights the importance of functional investigation of the microbial abundance at single-cell resolution for understanding their immune-cell-specific behavior and potential contribution to exacerbating disease outcomes.

Intracellular microbes employ unique resistance strategies. To treat these intracellular microbes, a variety of antibiotic classes, including β-lactams, macrolides, quinolones, aminoglycosides, tetracyclines, and more, are used.^53^ These antibiotics target various processes to inhibit microbial growth. However, a persistent challenge lies in the ability of antibiotics to effectively reach and remain active within the host cells, particularly within subcellular-membrane-protected organelles where intracellular bacteria can conceal.^54^ Additionally, several intracellular microbes have developed antibiotic resistance as a defense mechanism to survive within the host cell. For instance, there is a concern that β-lactamase genes found in Streptomyces species might potentially transfer to other pathogenic bacteria present in the vicinity.^55^ On the other hand, *S. aureus* has demonstrated the capability to develop resistance against a wide range of clinically available classes of antibiotics. This resistance can arise through mutations in the chromosomal genes or the acquisition of horizontally transferred resistance determinants.^56^ Among the 76 microbes detected in our study, several of them are reported to have resistance against commonly used antibiotics (Table S5). The presence of antimicrobial resistance genes within these intracellular microbes, often classified as non-pathogenic, raises significant interest. These microbes possess the ability to transfer resistance genes horizontally to pathogenic strains, potentially exerting diverse health effects.^57^^,^^58^ Consequently, it becomes imperative to employ precise methods for the detection and quantification of these intracellular microbes.

scRNA-Seq is emerging as a powerful tool for understanding the complexity of RNA transcripts within individual cells. In our study we used this technique to uncover the complex interplay between intracellular microbes and their interactions with host immune cells in healthy, COVID-19-positive, and recovered individuals. Herein, we found that the immune cells harbor diverse intracellular microbes. This diversity correlated with the significant physiological changes of the body. Interestingly, we found presence of *B. aphidicola*, *S. clavuligerus*, and *E. canis* in memory B cell, naive T cell, and Treg cell, as they typically do not harbor intracellular microbes. Although we observed a higher microbial abundance in the antigen-presenting cells, differential abundance of microbes in other immune cells needs attention. Our study also highlighted six bacteria—*E. canis*, *B. aphidicola*, *S. clavuligerus*, *S. pristinaespiralis*, *P. tolaasii*, and *E. albertii*—as being of importance at the cellular level and should be looked closely in future studies for their role in modulating disease and recovery. However, despite finding these intriguing microbial footprints within the immune cells, a significant question remains shrouded in uncertainty about the precise role played by these microbes within these immune cells, which awaits further rigorous investigation. Resolving this enigma promises to deepen our insight into the complex crosstalk between intracellular microbes and the host cells, unveiling potential approaches for therapeutic interventions and novel approaches to prevent infections.

### Limitations of the study

Detailed functional investigation of the cell-type-specific role of these microbes in the disease context was not performed in the present study, which is an important area of future research. A follow-up study investigating the same not only in PBMCs but also in other tissue types is warranted. Furthermore, although our study focused on COVID-19, we encourage similar research to be done in the context of other infectious diseases. This broader approach can enhance our understanding of the roles of these microbes in various disease contexts and contribute to the advancement of therapeutic interventions.

## STAR★Methods

### Key resources table

**Table:** 

REAGENT or RESOURCE	SOURCE	IDENTIFIER
TRUPCR® SARS-CoV-2 RT qPCR Kit	3B BlackBio	Cat# 3B306
Ligation sequencing kit	Oxford Nanopore Technologies	Cat# SQK-LSK109
Native barcoding expansion kit	Oxford Nanopore Technologies	Cat# EXP-NBD104
BD Vacutainer® CPT™	Becton Dickinson	Cat# 362753
BD Human single-cell multiplexing kit	Becton Dickinson	Cat# 633781
BD Rhapsody WTA amplification kit	Becton Dickinson	Cat# 633801
BD Rhapsody cDNA kit	Becton Dickinson	Cat# 633773
AMPure XP	Beckman Coulter	Cat# A63881
Qubit dsDNA HS Assay kit	Invitrogen	Cat# Q32854
Agilent 2100 Bioanalyzer	Agilent	Cat# 5067-4626
NovaSeq 6000 S2 reagent kit (200 cycles)	Illumina	Cat# 20040326

**Deposited data**		

Raw and analyzed data single-cell data	This paper	GEO: GSE201088
Raw bulk RNAseq data	This paper	SRA: PRJNA816679; https://www.ncbi.nlm.nih.gov/bioproject/?term=PRJNA816679

**Software and algorithms**		

bcl2fastq 2.19	NA	GitHub - brwnj/bcl2fastq: NextSeq specific bcl2fastq2 wrapper.
Kraken2 2.1.2	Wood et al.^59^	https://github.com/DerrickWood/kraken2
Pavian 1.2.0	Breitwieser et al.^60^	https://github.com/fbreitwieser/pavian
metagenomeSeq 1.42.0	Paulson et al.^61^	https://github.com/HCBravoLab/metagenomeSeq
phyloSeq 1.44.0	McMurdie et al.^62^	https://github.com/joey711/phyloseq
STAR 2.5.2b	Dobin et al.^63^	https://github.com/alexdobin/STAR/releases
Seurat 4.2.0	Hao et al.^64^	https://github.com/satijalab/seurat
BD rhapsody WTA analysis pipeline	NA	https://www.bdbiosciences.com/content/dam/bdb/marketing-documents/BD_Single_Cell_Multiomics_Analysis_Setup_User_Guide.pdf
CellMarkerDB	Zhang et al.^65^	http://biocc.hrbmu.edu.cn/CellMarker
PanglaoDB	Franzen et al.^66^	https://panglaodb.se/index.html
Azimuth	Hao et al.^67^	https://azimuth.hubmapconsortium.org/
Trimmomatic v0.39	Bolger et al.^68^	https://github.com/usadellab/Trimmomatic
ggpubr v0.6.0	Kassambara et al.^69^	https://github.com/kassambara/ggpubr
ggplot2 v3.4.2	Wickham et al.^70^	https://github.com/tidyverse/ggplot2
TBtools v1.123	Chen et al.^71^	https://github.com/CJ-Chen/TBtools-II/releases
Prism 9	GraphPad	https://www.graphpad.com/
Rawgraphs 2.0 beta	Mauri et al.^72^	https://app.rawgraphs.io/

### Resource availability

#### Lead contact

Further information and requests for resources and reagents should be directed to and will be fulfilled by the lead contact, Rajesh Pandey (rajesh.p@igib.res.in).

#### Materials availability

This study did not generate new unique reagents and material.

#### Data and code availability

• scRNA-seq data have been deposited at NCBI GEO repository and are publicly available as of the date of publication. Accession numbers are listed in the key resources table. All the data reported in this paper will be shared by the lead contact upon request.
• This paper does not report any original code.
• Any additional information required to reanalyze the data reported in this paper is available from the lead contact upon request.

### Experimental model and study participant details

#### Human subjects and clinical protocol

The samples were collected from Dr. D. Y. Patil Medical College, Hospital and Research Institute in Kolhapur, Maharashtra, India. Healthy volunteers, patients with confirmed COVID-19 positive, and patients who had recovered from COVID-19 were included in the study. The age and gender distribution are available in Table S1. In this study, the median age was 23 years for the Healthy participants, 62 years in COVID-19 patients and 34 years in the Recovered group. The gender (M/F ratio) among the three groups was comparable. All recovered samples were collected within a month of recovery and confirmed by negative RT-PCR (TRUPCR® SARS-CoV-2 RT qPCR Kit, catalog no 3B306, Ct value > 35). Oxford Nanopore sequencing was used to identify the SARS-CoV-2 variant that was causing infection (Ligation sequencing kit, catalog no SQK-LSK109, Native barcoding expansion kit, EXP-NBD104). 13 out of 16 people in the COVID-19-positive group had the 20B version, and the remaining three had the 20A variant. 11 out of the recovered group's 13 members had the 20B infection, while the remaining two had the 20A infection. The samples were anonymized, and the comprehensive clinical and demographic data were recorded electronically. Institutional ethical clearance for the study was obtained from both CSIR-IGIB and the Dr. D. Y. Patil Medical College, Hospital and Research Institute. The studies involving human participants were reviewed and approved by CSIR-IGIB’s Human Ethics Committee Clearance (Ref No: CSIR-IGIB/IHEC/2020-21/01). The study was conducted following the guidelines of the Declaration of Helsinki. The patients/participants provided their written informed consent to participate in this study.

#### Collection and classification of clinical samples

Blood samples were collected using BD Vacutainer CPT cell preparation tubes that contained sodium heparin. PBMCs were isolated from 5 ml of blood using a BD Vacutainer CPT™ cell preparation tube with sodium heparin. The method involved centrifugation at room temperature (RT) with a speed of 1800 RCF for 20 minutes, followed by two washes with PBS at 300 RCF for 15 min at RT. The preserved PBMCs were cryopreserved in a media composed of 90% fetal bovine serum (FBS) and 10% dimethyl sulfoxide (DMSO) to maintain their long-term viability and integrity for future analysis. The patients were categorized into groups based on the results of the real-time reverse transcription-polymerase chain reaction (RT-PCR) test for SARS-CoV-2 infection. These groups include Healthy individuals, those who tested positive for COVID-19, and individuals who had Recovered (RT-PCR negative) from the infection at least a month prior sample collection.

### Method details

#### Sample processing and library preparation and sequencing

The PBMCs were revived and processed using the BD Rhapsody single cell analysis system, following the protocol described by Chattopadhyay et al. Briefly, 0.2 million cells per sample were taken and labeled using the BD™ Single-Cell Multiplexing Kit-Human (catalogue no 633781), according to the manufacturer's guide (Doc ID: 214419 Rev. 2.0). An average of 30,000 pooled cells were loaded in each cartridge on the BD Rhapsody express single cell analysis system for single cell capture, followed by cDNA synthesis (BD Rhapsody cDNA kit, catalogue no 633773), as per the manufacturer's guideline (Doc ID: 210967 Rev. 1.0). The mRNA Whole Transcriptome Analysis (WTA), and Sample Tag library were prepared using the BD Rhapsody™ WTA Amplification kit (catalogue no 633801), according to the manufacturer's guideline (Doc ID: 23-21752-00). The libraries were checked for their quality using Qubit dsDNA HS Assay kit (Invitrogen, catalogue no Q32854) and Agilent 2100 Bioanalyzer (catalogue no 5067-4626). The libraries were then sequenced using the NovaSeq 6000 S2 reagent kit (catalogue no 20040326), with 30,000 reads/cell for WTA, and 120 reads/cell/Sample Tag for sample tag library, with 101 x 2 cycles.

#### Data demultiplexing and metagenomic analysis

The raw sequencing data was demultiplexed and converted to FASTQ format using the bcl2fastq tool.^73^ The demultiplexed reads were aligned to the human reference genome (GRCh38). The unmapped reads were checked for the presence of microbial reads using Kraken2 tool using Refseq references including archaea, bacteria, viruses, fungi, and protozoan genomes for classification of unmapped reads.^59^ The kraken output files were visualized using the Pavian tool to generate the count matrix for microbial abundance.^60^ Next CSS normalization was used on the microbial abundance data using metagenomeSeq R package.^61^ The genus with <1% abundance were filtered out using the phyloseq R package.^62^ The corresponding species level abundances were also checked for abundance >0.1% and 76 microbial species were selected for further single-cell-level analysis (see below figure).

**Figure:**
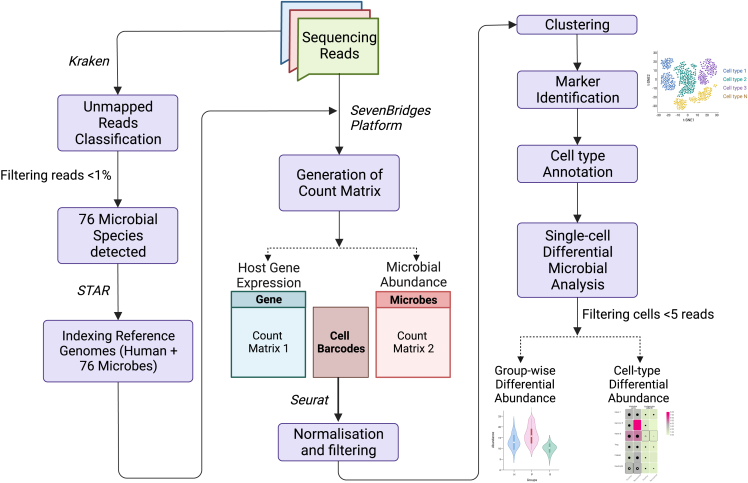
A flowchart illustrating the steps taken for analyzing the differences in microbial abundance at a single-cell resolution from scRNA-seq data

#### scRNA-seq data analysis, clustering and cell type annotation

A manually curated reference genome index was generated using STAR aligner for single-cell analysis,^63^ human genome (using GRCh38) and the genome references of 76 microbes identified from Kraken were included (Table S6). The fastq files were processed using the BD Rhapsody WTA analysis pipeline as per the manufacturer’s guideline (Doc ID: 47383 Rev. 9.0). The count matrix with substitution error correction was imported to the Seurat R package for downstream analysis and visualization.^64^ A total of 109099 cells were merged from healthy, COVID-19 positive, and recovered patients for integrated multimodal analysis. Cells with between >20 and <2500 UMI were retained, and clustering was performed using an unsupervised method at a resolution of 0.4 and visualized with tSNE algorithm. Cluster specific genes were identified using FindAllMarker function of seurat R package (Wilcoxon rank sum test, log2 fold change cut-off 1.5) for comprehensively annotating clusters with cell type. The clusters were identified manually using CellMarkerDB and PanglaoDB, as well as automated annotation by Azimuth.^65^^,^^66^^,^^67^

#### Alpha and beta diversity analysis

From the annotation, the microbial abundance count matrix was group-wise and cell-type specific to perform differential abundance analysis. The Shannon alpha diversity and Beta diversity (using Bray Curtis distance matrix) was calculated for the microbial reads using the phyloseq R package.^62^ The Principal Component Analysis (PCoA) was used to visualize beta diversity and an adonis test was performed to check for significance.^74^^,^^75^

#### Metagenomic analysis of bulk RNAseq data

The ICU-admitted COVID-19 patients PBMCs were used from BioProject PRJNA816679, sample IDs mentioned in Table S7. The raw fastq files were trimmed using Trimmomatic, to filter out low quality reads and bases and adapter contamination.^68^ The processed reads were analyzed similarly to scRNAseq data as mentioned before. Kraken2 was used for classifying reads unmapped to the human genome. The classified reads were normalized using the CSS normalization method of metagenomeSeq R package and a cut off of >0.1 was applied to select microbial reads.

#### Differential microbial abundance analysis

The cells subset with > 5 reads were included for differential microbial abundance analysis. The group-wise comparison was performed using normalized microbial abundance counts for all the samples compared using Mann-Whitney U test. The cell-type specific group wise comparisons were made using FindMarkers function in Seurat (using Wilcoxon Test). The cell types with counts > 47 in each group were considered for comparisons. The microbes with p adjusted value <0.05 and with presence in more than 10% cells in at least one group were considered as significant. The data was visualized using ggpubr and ggplot2 R packages Tbtools.^69^^,^^70^^,^^71^ All the statistical tests were two-tailed and p value <0.05 was considered as significant unless mentioned otherwise. Rawgraphs 2.0 was also used for data visualization.^72^

### Quantification and statistical analysis

Mann-Whitney U test was used to compare the microbial read counts (Figure 1D), cell proportions (Figure 2B), differential microbial abundance between groups (Figures 2F–2U) and to compare the microbial abundances between bulk and single-cell RNAseq (Figures 4E–4L). The Kruskal Wallis test used to calculate diversity richness significance of alpha diversity (Shannon) (Figures 2C and 2D), while Adonis test was used to acquire beta diversity significance (Figure 4B). The Wilcoxon rank sum test was used to identify significant cluster specific genes (Figure 1B) and compare cell-type specific group wise comparisons (Figure 3C). The data visualization and statistical analysis were performed in licensed version of GraphPad prism 9 and R V4.2.0 along with the following packages: phyloseq, ggplot2, ggpubr, Rawgraphs 2.0, Tbtools and metagenomeSeq R. All the statistical tests were two-tailed and p value <0.05 was considered as significant.

## Supplemental information

Table S1. Clinical Parameters of patient cohort for scRNAseq, related to Figure 1

Table S2. Phylum, genus, and species with >0.1 abundance across three groups, related to Figure 2

Table S3. Cell-type-specific microbial abundance, related to Figure 3

Table S4. Sample-wise relative abundance of species (>0.1%) in bulk RNAseq COVID-19-positive cohort, related to Figure 4

Table S5. Known antimicrobial resistance among the detected bacterial species, related to Figure 5

Table S6. Accession Id of the bacterial species used for further single-cell analysis, related to Figure 6

Table S7. Accession Id of the samples from bulk RNA-seq of COVID-19-positive patients, related to Table 1
